# Navigating Care: Jockey Club Carer Space Project and Its Impact on Developing a Carer-Friendly Community in Hong Kong

**DOI:** 10.1093/geroni/igaf122.032

**Published:** 2025-12-31

**Authors:** Vivian Lou, Vera Mun, Yu Tang, Sau Man, Angela Luk, Tarani Chandola, Wai Sze Chan, Jianchao Quan, Terry Lum

**Affiliations:** The University of Hong Kong, Hong Kong, China (People’s Republic); The University of Hong Kong, Hong Kong, China (People’s Republic); Caritas, Hong Kong, Hong Kong, China (People’s Republic); The University of Hong Kong, Hong Kong, China (People’s Republic); The University of Hong Kong, Hong Kong, China (People’s Republic); The University of Hong Kong, Hong Kong, China (People’s Republic); The University of Hong Kong, Hong Kong, China (People’s Republic)

## Abstract

The Jockey Club Carer Space Project aims to enhance the well-being of carers of older adults in Hong Kong through community-centered research and support. Grounded in community-engaged scholarship, the project employs participatory co-creation methods, engaging community stakeholders in all stages of research, service model development and intervention. Key findings include improved caregiver well-being, reduced stress, and increased confidence. The project has also developed innovative methodologies that prioritize community input, enhancing the relevance and effectiveness of interventions. Policy implications highlight the need for supportive measures such as flexible respite care, and carer-centric design. This initiative exemplifies the impact of collaborative, community-driven research in creating sustainable solutions for carer-friendly communities.

